# The Relationship of Policymaking and Networking Characteristics among Leaders of Large Urban Health Departments

**DOI:** 10.3390/ijerph120809169

**Published:** 2015-08-06

**Authors:** Jonathon P. Leider, Brian C. Castrucci, Jenine K. Harris, Shelley Hearne

**Affiliations:** 1 de Beaumont Foundation, 7501 Wisconsin Avenue, Suite 1310E Bethesda, Maryland, MD 20814, USA; E-Mail: castrucci@debeaumont.org; 2 Brown School, Center for Public Health Systems Science, Washington University in St. Louis, Missouri, MO 63130, USA; E-Mail: harrisj@wustl.edu; 3 Director, Big Cities Health Coalition, National Association of County and City Health Officials, 1100 17th Street, NW, Seventh Floor, Washington, DC 20036, USA; E-Mail: shearne@naccho.org

**Keywords:** public health systems, network research, big cities health coalition, urban health, policy networks

## Abstract

Background: The relationship between policy networks and policy development among local health departments (LHDs) is a growing area of interest to public health practitioners and researchers alike. In this study, we examine policy activity and ties between public health leadership across large urban health departments. Methods: This study uses data from a national profile of local health departments as well as responses from a survey sent to three staff members (local health official, chief of policy, chief science officer) in each of 16 urban health departments in the United States. Network questions related to frequency of contact with health department personnel in other cities. Using exponential random graph models, network density and centrality were examined, as were patterns of communication among those working on several policy areas using exponential random graph models. Results: All 16 LHDs were active in communicating about chronic disease as well as about use of alcohol, tobacco, and other drugs (ATOD). Connectedness was highest among local health officials (density = .55), and slightly lower for chief science officers (*d* = .33) and chiefs of policy (*d* = .29). After accounting for organizational characteristics, policy homophily (*i.e.*, when two network members match on a single characteristic) and tenure were the most significant predictors of formation of network ties. Conclusion: Networking across health departments has the potential for accelerating the adoption of public health policies. This study suggests similar policy interests and formation of connections among senior leadership can potentially drive greater connectedness among other staff.

## 1. Introduction

Three of the ten essential services of public health articulate the role of governmental public health in developing policy [1,2,3]. To provide these essential services, local health departments (LHDs) prepare issue briefs, give testimony, participate on advisory boards, communicate with policymakers, and provide technical assistance in policy creation [4,5]. Participation in these activities varies significantly by health department jurisdictional size, with large health departments conducting more policy activities than their smaller counterparts [6]. 

In a recent comparison of policy activity between the members of the National Association of County and City Health Officials’ (NACCHO) Big Cities Health Coalition (BCHC)—a group of urban health leaders from the largest LHDs in the United States—and members of health departments of different jurisdictional sizes, Hearne *et al.*, found that all BCHC members (100%) reported conducting at least one of the five policy activities tracked by NACCHO in the past year at the local level, compared to 81% of personnel at all LHDs [7]. Seventy-five percent of BCHC members participated in at least one area at the federal level, compared with 33% of other large LHDs serving 500,000 persons or more, and 15% of all LHDs nationwide [7]. In addition to depth of policy involvement, recent research has also shown urban health departments are involved in more types of policy—health and otherwise—in their jurisdictions [6,7,8,9,10,11]. Networks have been shown to improve LHD performance [12,13,14,15] and can make the policy process more efficient and innovative [16]. The BCHC has purposefully created a network of the largest health departments in the United States with a key objective of leading the development, adoption, and implementation of public health policy nationwide [17,18,19]. Their stated purpose makes the group an appropriate point of inquiry around policy-related network issues. 

The aims of this study are two-fold. First, the study seeks to describe the network of connections among the BCHC membership. Second, the study seeks to explore the relationship between this network and policy activity. These questions add to the discussion of the importance of a connected social network of health department leaders for policy innovation and advocacy, both within local jurisdictions and nationwide.

## 2. Methodology

### 2.1. Two Datasets

This manuscript draws on two primary sources of information. The first is the 2013 NACCHO Profile. The Profile represents a census of all 2800 LHDs in the country, condensed into 2532 reporting units. The Profile is conducted approximately biennially, and historically has high response rates. In 2013, the response rate was 78% overall, and 17 of 18 BCHC members participated in the Profile [4]. The Profile encompasses several areas of health department characteristics, including size of population served, staff size, budget, activities conducted, policy activities, and other infrastructure-related components. The Profile represents the only systematic and longitudinal source of information about all the nation’s LHDs, and has been conducted since the late 1980s [5]. We utilized several questions related to policy activity and collaboration. 

The second dataset used in this study comes from a 2013 survey of leaders of the BCHC conducted by the authors; these data focus on communication and collaboration among BCHC members [7]. Forty-seven key LHD staff members participated. In each jurisdiction, we attempted to survey staff in three positions: the local health official (LHO), the chief of policy/senior deputy (CP/SD), and the chief science officer, or equivalent (three jurisdictions did not have chiefs of policy). These positions were selected specifically for their respective roles in the organization around leadership and technical expertise. At the time this study was conducted, 18 LHDs constituted the BCHC. Nine more have since joined. The 18 member health departments were Atlanta, Baltimore, Boston, Chicago, Cleveland, Dallas, Denver, Detroit, Houston, Los Angeles, Miami, New York, Philadelphia, Phoenix, San Francisco, San Jose, Seattle, and Washington, DC. 

### 2.2. The Survey

Before the survey was fielded electronically directly to the selected LHD staff members, it was pretested with five former local public health officials. One section of the broader survey focused on collaboration and communication with other BCHC members. Respondents were asked how frequently they interacted with their peers in a particular BCHC jurisdiction (*i.e.*, LHO with LHO, chief of policy with chief of policy, chief science officer with chief science officer)—weekly, monthly, yearly, less than yearly, or whether they did not know their peer in that jurisdiction.

### 2.3. Contact Network Definitions

“Contact networks” were created by constructing a dataset representing the relationships between staff across different LHDs in terms of frequency of contact. For analysis, the contact networks were dichotomized so that a tie was shown between two network members for any level of contact, whereas no tie was shown for no contact. Because contact between two people is an inherently non-directional relationship, we symmetrized the network. Dyads in which one partner indicated no contact and the other indicated contact were considered linked. Dyads in which data was missing for one of the two partners took the value submitted by the responding partner. Dyads with both partners missing, or both partners indicating no contact, were considered unlinked.

### 2.4. Network Density and Communication Patterns

We examined network density and identified central network members using betweenness centrality, which measures how often a network member provided a bridge between two other network members who were not linked. We also examined patterns of ties among those working on policy in the top three areas in which BCHC members had passed new ordinances in the past year: environmental, tobacco alcohol and other drugs (ATOD), and chronic disease/obesity. Specifically, we developed exponential random graph models (ERGM) predicting the likelihood of a tie between two health departments. ERGM is similar to logistic regression in predicting the probability of a tie between any two network members while accounting for the underlying assumption that links in a network are not independent [20]. Like terms in a logistic regression model, odds ratios for terms in an ERGM indicate an increase or decrease in the odds of a tie between two network members with the given characteristic. We developed separate ERGMs for LHOs, epidemiologists, and chief of policy/senior deputies to predict the probability of a link between two network members based on: (1) a binary variable indicating whether the LHOs of the two health departments were connected (CSO and chief of policy/senior deputy only); (2) the number of years of employment of the LHO, CSO, or CP/SDs; and (3) whether the two LHDs had both passed (or not passed) each of three policies related to chronic disease/obesity, environmental health, or use of alcohol, tobacco, or other drugs (ATOD). Two network members matching on a characteristic is called homophily (e.g., similar organization type) and is common in observed networks [20], including networks of LHDs [21]. Specifically, terms indicating whether a link was more likely between two LHDs that had both passed (or not passed) a given policy are *policy homophily* terms. To summarize, each model predicted the probability of a tie between two network members based on how long each network member had been employed, whether the leaders of the two health departments were linked, and whether both health departments conducted activities in the same policy areas.

## 3. Results

### 3.1. Policy Activity

**Figure 1:**
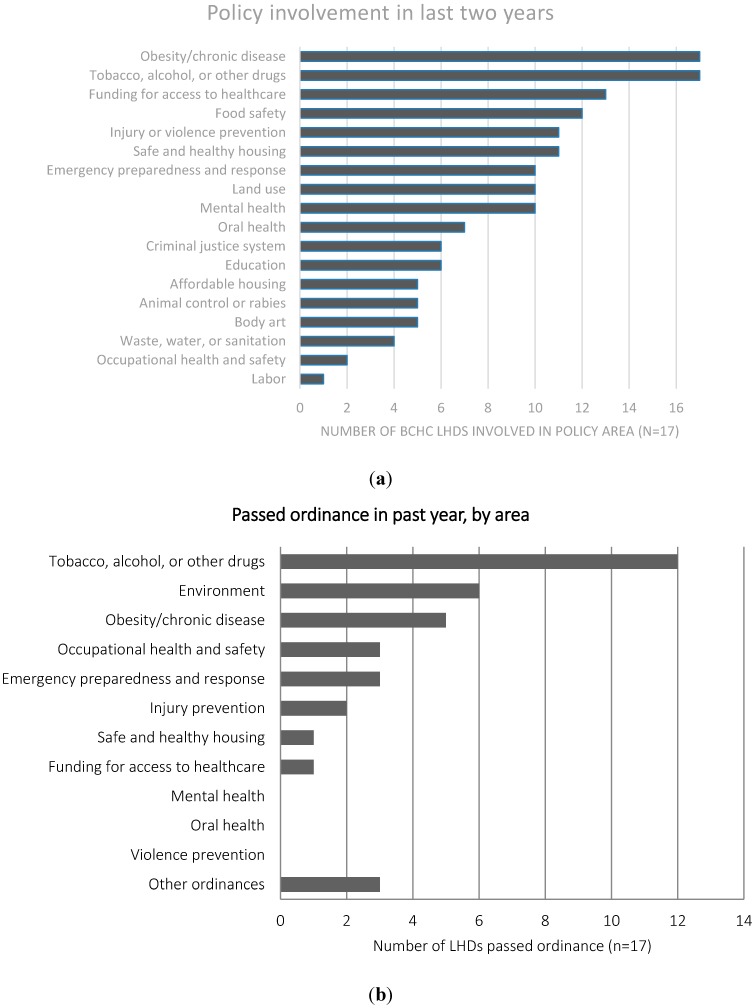
Policy involvement (**a**) and passed ordinances (**b**) among local health departments (LHDs) in the Big Cities Health Coalition (BCHC). Note: These figures illustrate the involvement of 17 large urban health departments in the United States in policy activities, as well as how many cities passed ordinances in particular health topics.

Respondents in all BCHC cities indicated they had worked on policy initiatives in obesity/chronic disease and tobacco, alcohol or other drugs (ATOD) in the past 2 years (Figure 1). In the chronic disease area, 75% said they were working on: (1) a school physical activity policy; (2) increasing health food options at schools and for the general public; and (3) urban policy design. Within ATOD policymaking, the majority of BCHC members worked on smoke-free indoor and outdoor air policies and reducing the sale of cigarettes to minors, with 6 of 18 members working on raising the cigarette tax. 

Local governments in 15 BCHC jurisdictions passed a local public health ordinance between 2011 and 2013. Twelve jurisdictions passed ATOD ordinances, 6 passed environmental ordinances, 5 passed obesity/chronic health ordinances, 3 passed occupational health ordinances, 3 passed emergency preparedness ordinances, 2 passed injury-prevention ordinances, 1 each passed ordinances related to housing and access to healthcare, and 3 passed some other ordinance. None of the BCHC members were in jurisdictions that passed ordinances related to mental health, oral health, or violence prevention. LHDs report to city and county governments that are responsible for passing these ordinances and are often involved in the conception and implementation of the ordinances.

### 3.2. Contact between BCHC Staff

**Figure 2:**
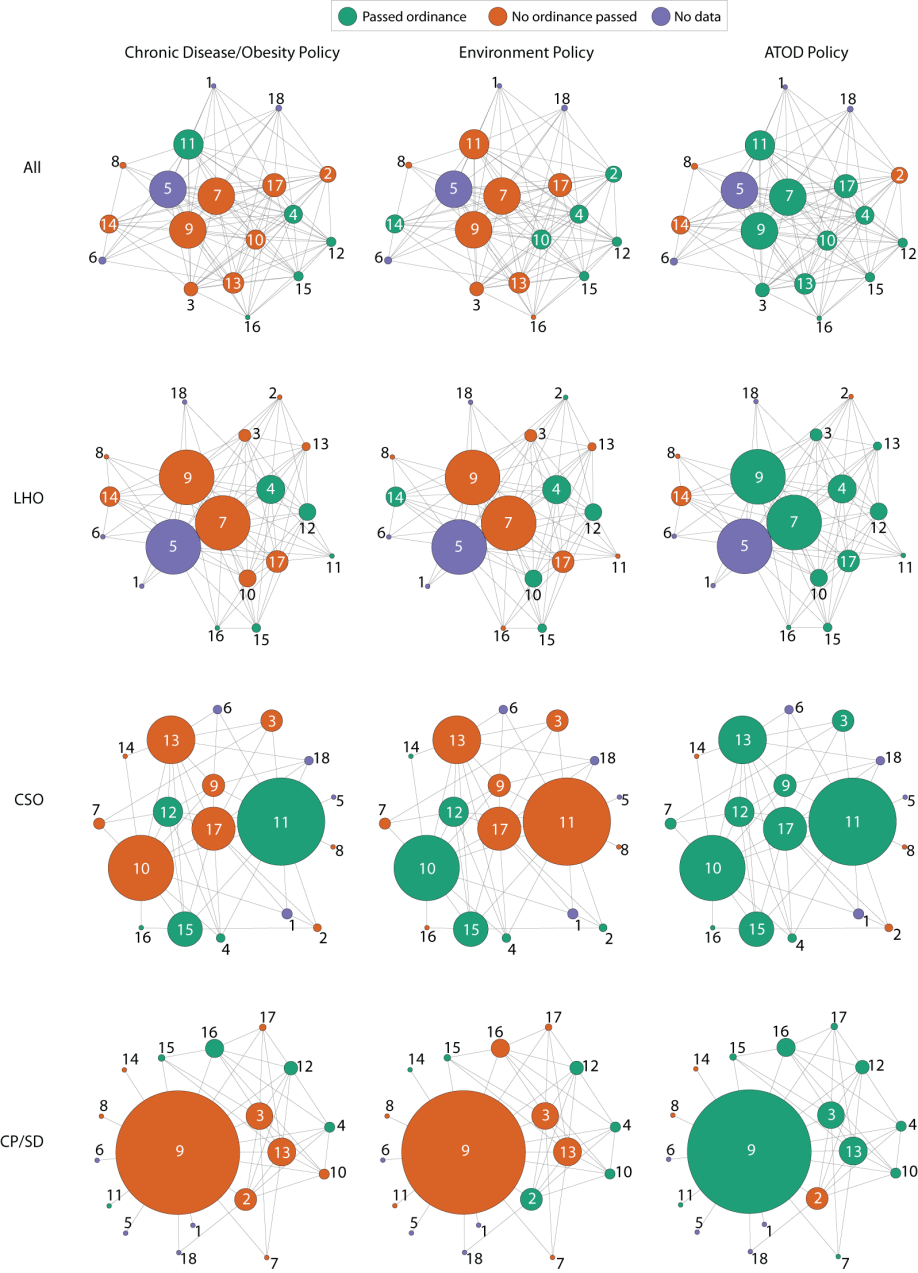
Ordinance adoption across the networks. Nodes sized by betweenness centrality within a policy area (column heading) and position type (row). ATOD: Alcohol, Tobacco, and Other drugs. LHO: Local Health Official. CSO: Chief Science Officer. CP/SD: Chief of Policy/Senior Deputy.

The majority of BCHC staff we contacted participated in the survey; response rates were 89% (16/18) of LHOs and CP/SDs participating and 83% (15/18) of CSOs. Each nonrespondent indicated a lack of time to participate in the study during followup. As part of the survey of BCHC leadership, participants were asked how frequently they contacted their peers across the various agencies. The overall network, including all ties between health departments, was very dense (*d* = .71). A density of 1.0 would mean each LHD staff member was connected to peers in every other LHD. For the three types of LHD professional (LHO, CP/SD, and CSO), network density was highest for LHOs, with 55% of possible ties in the network (*d* = .55). Density was lower for chief science officers, with 33% of possible ties (*d* = .33) and lowest for chiefs of policy/senior deputies, with 29% of possible ties (*d* = .29). Figure 2 shows the networks overall as well as for each type of LHD professional in each of the three policy areas. 

The size of the nodes in these graphs represents betweenness centrality, or how often the network member acts as a bridge between other LHDs who are not directly connected. Betweenness centralization is an indicator of how centralized a network is around a single network member who is acting as a bridge to connect different parts of the network together. When one network member has very high betweenness centrality compared to most or all of the other network members, the betweenness centralization of the network will be high. Centralization was least for the overall network (*b* = .04), followed by, in ascending order, the LHO network (*b* = .11), the chief scientist network (*b* = .31), and the chiefs of policy/senior deputy network (*b* = .68). For example, in the CP/SD network, health department 9 connects 6 other LHDs to the network. Therefore LHD 9 has high betweenness (shown by its very large node) in the network of senior deputies and may have significant influence over how information spreads through this network. Likewise, the CSO in LHD 11 and the LHO in health departments 6, 7, and 9 are all central to their networks. 

To determine the influence of ties between LHD leaders *(i.e.*, LHOs) on ties between other types of LHD employees, we first examined a simple network model including only the constant term accounting for the number of ties in the network (edges) and including a predictor for whether having a tie between two LHOs changed the likelihood of a tie between CSOs or senior deputies at the same two LHDs. We found that CSOs at two different health departments were no more likely to have contact with each other if their leaders were in contact (*OR* = 1.74; 95% *CI*: 0.87–3.49); however, senior deputies at two different health departments were more likely to be connected if their leaders were connected (*OR* = 6.97; 95% *CI*: 2.86–17.02). 

We added years of employment at the LHD and policy homophily terms to the simple models. The magnitude, direction, and significance of the leadership predictors remained consistent (Table 1). Years of employment, dichotomized to 2 years or less and more than 2 years, was positively and significantly associated with forming ties for all three networks. LHOs with more than 2 years of experience had 1.74 higher odds of forming ties as those with 2 years or less. Similarly, chief science officers and chiefs of policy/senior deputies with more years of experience had 2.05 and 2.71 higher odds of forming ties with other health department staff, respectively, compared to those with less experience. 

Policy homophily was positively and significantly associated with tie formation for ATOD. Nearly all of the LHDs in the network had passed ATOD policy, so this is logical. However, policy homophily was not significantly associated with tie formation for environmental policy or obesity/chronic disease policy, indicating that two health departments were no more or less likely to connect if they shared this policy characteristic.

**Table 1:** Exponential random graph models (ERGM) predicting the likelihood of a tie between LHOs, CSOs, and SDs in two large local health departments.

	LHOs OR (95% CI)	CSO OR (95% CI)	Sr Dep OR (95% CI)
Edges (constant)	0.31 (0.13–0.72) *	0.14 (0.06–0.31) *	0.02 (0.01–0.08) *
LHD leadership linked	Na	0.87 (0.38–1.98)	5.91 (2.11–16.59) *
>2 years current position	1.74 (1.01–3.01) *	2.05 (1.15–3.66) *	2.71 (1.35–5.44) *
Policy homophily			
ATOD	4.13 (1.83–9.29) *	5.48 (2.28–13.17) *	3.10 (1.24–7.71) *
Environmental	0.89 (0.38–2.10)	0.76 (0.31–1.84)	1.34 (0.52–3.47)
Obesity/Chronic	1.93 (0.84–4.44)	0.93 (0.40–2.17)	2.09 (0.85–5.11)

Note: * indicates statistical significant at *p* < .05

## 4. Discussion

In the past several years, the majority of BCHC member leadership has been forming connections. This is occurring in the context of national changes in policy, such as passage of the Patient Protection and Affordable Care Act and transfat bans, as well as local initiatives, such as e-cigarette regulation, overdose prevention and treatment, and addressing the obesity epidemic [22,23,24,25,26]. The BCHC networks were extremely dense, which counters a recent study’s findings of low network density in the national network of all LHDs, but is consistent with the finding that health departments of similar size tend to connect to each other [21]. Consistent with much network research, we found density and centralization were inversely related in the BCHC contact networks [27]. As network density decreased, network centralization increased. Density and centralization have been positively related to effectiveness in policy networks [21]. LHO participation was strongly associated with linkage among senior deputies, suggesting that the benefits of affinity groups like the BCHC possibly extend beyond those directly engaged. However, if creating interdepartmental networks is important, more opportunities for direct peer-to-peer exchange must be created in staff positions other than the LHO.

Also consistent with past research [21], the length of time staff spent in their current position was positively and significantly associated with forming ties for LHOs, CSOs, and CP/SDs, demonstrating the influence of staff tenure on relationship building. With forecasts suggesting significant turnover in the public health workforce [28,29,30,31,32], especially among leadership levels, strategies are needed that will engage those who are new in their positions and those who may be identified as potential future senior leaders to create deliberate opportunities for them to build networks [21,33].

We also explored the link between social networks and policy adoption, finding an association for ATOD policies but not for either obesity/chronic or environmental policies. A possible explanation of links associated with ATOD may be related to the long history of tobacco control in urban health departments [34,35,36,37]. This includes direct categorical funding that supported and encouraged networking through conferences, creating a network that is “intentionally grown.” Conversely, for other policy areas, networks are more “organically grown” and are perhaps slower to develop. Building on this, a strategy for network growth may be to create “intentionally grown” networks (e.g., centers for excellence in a city, staffing to promote networks around a specific topic) to accelerate translation.

This study suggests that increased interorganizational networking could be a possible strategy to promote policy change at the local level, but understanding why this association exists for one type of policy and not others is beyond the scope of this study. However, past studies have found that LHDs with denser networks to have better performance [14,15], and that policy networks with higher density and more centralization are more efficient [16]. Further research is needed to understand the role of these interorganizational networks in local and national public health policy adoption.

### Limitations

This study has several limitations. LHDs tend to connect with other LHDs that share a border or are in the same state; however, given the geographical dispersion of the BCHC members, we did not include an indicator of geographic proximity in the model. Non-response may be an issue, although response rates are high enough to allow for robust network analysis. An additional consideration is generalizability to other large LHDs. Since the time of this study, the number of BCHC members has increased. It may be that networks are only beginning to form between new members and existing ones. 

## 5. Conclusions

This study demonstrates the importance of leadership in creating interorganizational networks. It also suggests that there may be a potential role for interorganizational networks in supporting policy. However, constricted public health agency budgets and increased limitations on Federal funds to support traditional networking events such as conferences and workshops can be barriers to establishing and developing networks. Further research to understand the relationship between interorganizational networks and policy may support increased funder investment in traditional and more innovative methods (e.g., personal connections and online communities) of network development. 
